# Self-limiting thoracic aortic dissection during bronchial artery embolization

**DOI:** 10.1590/0100-3984.2015.0216

**Published:** 2017

**Authors:** Rafael Dahmer Rocha, Joaquim Maurício da Motta-Leal-Filho, Francisco Leonardo Galastri, Breno Boueri Affonso, Humberto Bogossian, Felipe Nasser

**Affiliations:** 1 Department of Interventional Radiology and Pulmonology, Hospital Israelita Albert Einstein, São Paulo, SP, Brazil.

**Figure 1:**
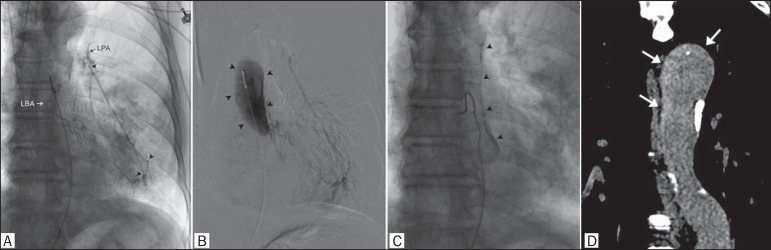
**A:** Left bronchial arteriography showing a shunt (arrowheads) between the left bronchial artery (LBA) and the left pulmonary artery (LPA). **B,C:** Stagnation of the contrast media (arrowheads) can be seen at the false lumen of the descending thoracic aorta, indicating dissection. **D:** Coronal CT reconstruction at 7 days after bronchial arteriography showing persistence of the contrast media in the false lumen of the thoracic aorta (arrows), with no increase in the extent of the dissection.

Dear Editor,

A 75-year-old woman presented with a 3-week history of intermittent hemoptysis related to a history of recurrent episodes of pneumonia. Chest computed tomography (CT) showed cylindrical bronchiectasis in the lingula, and bronchoscopy showed clots in the left bronchial tree. Bronchial arteriography was requested and revealed a shunt (Figure 1A) between the left bronchial artery and the left pulmonary artery. During manual-injection digital subtraction angiography, enhancement and stagnation of the contrast media were observed in a false lumen of the descending thoracic aorta (Figures 1B and 1C), consistent with iatrogenic aorta dissection. The iatrogenic aortic dissection extended to the left bronchial artery, leading to obstruction of blood flow to the shunt. However, there were no signs of hemodynamic instability, and the patient therefore received conservative therapy with clinical and radiological monitoring. A second CT scan, obtained 7 days later, showed that the iatrogenic aorta dissection was stable (Figure 1D), and a third scan, obtained 5 months later, showed total resolution. During 7 months of follow-up, the patient reported no pain or new episodes of bleeding. 

During endovascular procedures, iatrogenic aortic dissection can occur when the tip of the catheter is pushed into the vessel wall during catheterization, as well as when high-pressure jets of contrast media are directed toward the vessel wall. Although uncommon, iatrogenic aortic dissection accounts for approximately 5% of all thoracic aortic dissections^(1,2)^. In a review article, Ittrich et al.^(3)^ showed rates of subintimal short segment dissection of the aorta during bronchial arteriography ranging from 1% to 6.3%^(4,5)^, although there are virtually no images of such dissections in the literature. There is no standard for the management of iatrogenic thoracic aorta dissection. Uncomplicated dissection of the descending thoracic aorta is a relatively benign process, and complete spontaneous resolution is observed in most cases. Pharmacological treatment to control pain and blood pressure is recommended, as is short-term follow-up with CT^(6-8)^.
